# Decision and dopaminergic system: an ERPs study of Iowa gambling task in Parkinson’s disease

**DOI:** 10.3389/fpsyg.2014.00684

**Published:** 2014-07-03

**Authors:** Daniela Mapelli, Elisa Di Rosa, Matteo Cavalletti, Sami Schiff, Stefano Tamburin

**Affiliations:** ^1^Department of General Psychology, University of PadovaPadova, Italy; ^2^Human Inspired Technologies Research Center, University of PadovaPadova, Italy; ^3^Department of Medicine, University of PadovaPadova, Italy; ^4^Department of Neurological and Movement Sciences, Neurology Section, University of VeronaVerona, Italy

**Keywords:** Iowa gambling task (IGT), dopaminergic system, frontal lobe, decision making, Parkinson’s disease (PD)

## Abstract

Recent researches reported behavioral and emotional impairment in Parkinson’s disease (PD), even in the earliest stages. This impairment affects also decision-making and learning processes. The Iowa gambling task (IGT) is commonly used to examine the decision-making capacity. The purpose of the present study was to investigate the neural correlates of feedback evaluation in the decision-making process into a learning context, using IGT and event-related potentials (ERPs) in a group of non-demented medicated PD patients. Fifteen PD patients and 15 healthy controls were recruited for the study. PD patients were administrated a basic neuropsychological assessment oriented to exclude cognitive impairments. Both groups underwent the computerized IGT during electroencephalography (EEG) registration. To analyse ERPs, continuous EEG data were epoched within a time-window starting 1000 ms before and ending 1000 ms after feedback presentation and averaged separately for positive (i.e., win condition) and negative (i.e., loss condition) feedbacks. Behavioral data revealed a significant lower performance of PD patients (*p* < 0.05) compared with the controls. While controls demonstrated a correct feedback evaluation, PD patients did not show any learning, selecting more disadvantageous decks even in the last part of task. Furthermore, ERPs results revealed that controls showed a significant difference (*p* < 0.05) in ERPs morphology recorded after the win and the loss conditions, suggesting that positive and negative feedbacks were differently evaluated and processed. PD patients showed a different pattern: their ERPs morphology was the same for positive and negative feedback. Interestingly, our ERPs results suggest that in PD patients an incorrect evaluation of context-relevant outcomes could be the reason of a poor performance in decision-making tasks, and could explain cognitive and behavioral problems related to impulse control disorder.

## INTRODUCTION

A definition of the term *decision-making* is not easy, because it represents one of the highest and most complex human abilities, that is classically included in the executive functions. According to Rogers (2011), decision-making is a complex process that *encompasses a range of functions through which motivational processes make contact with action selection mechanisms to express one behavioral output rather than any of the available alternatives*. This definition implicitly assumes that the decision process is based on the functions of selection and inhibition, working memory, planning, emotion, estimation, and every process included in the term *executive control*.

Research about decision-making has largely increased within cognitive neuroscience over the last 20 years, starting from the study of patients with frontal lobe damage (Bechara et al., 1994, 1996; Damasio, 1994), to the emergence of new disciplines, such as neuroeconomics (Glimcher et al., 2008). Even though this increasing interest has been accompanied by the development of divergent models, a consensus has been reached concerning some of the fundamental aspects of decision-making. From a cognitive psychology perspective, decision-making can be considered as the integration of three complementary abilities: choice evaluation, response selection, and feedback processing (Fang et al., 2009). Feedback processing plays a central role in decision-making, because assigning a positive or negative valence to an option on the basis of previous experience is the prerequisite for the evaluation of our action outcomes and their anticipation and for an efficient response selection. The anatomical network underlying decision-making processes includes the prefrontal cortex (PFC), the anterior cingulate cortex (ACC), the fronto-striatal and limbic loops, and some subcortical structures (basal ganglia, amygdala; for a comprehensive review, see Gleichgerrcht et al., 2010).

Decision-making impairment has been documented in many different clinical conditions involving this network, mainly when PFC is damaged, including patients with frontal lobe damage (Bechara et al., 1996, 1997; Fellows and Farah, 2005), or with frontotemporal dementia (Rahman et al., 1999, 2005; Torralva et al., 2007, 2009). Healthy aging may affect the decision-making (Yates and Patalano, 1999; Finucane et al., 2002; MacPherson et al., 2002; Kovalchik et al., 2005; Cauffman et al., 2010; Eppinger et al., 2011) probably through slight changes in the functioning of this network.

Among the complex neuropharmacology of this anatomical network, dopamine (DA) is the main neuromodulator of the fronto-striatal loop, and plays a key role (Assadi et al., 2009; Rogers, 2011), in particular in reward processing during reinforcement learning (Schultz, 2002; Frank et al., 2004) and in learning and outcome monitoring (Hämmerer and Eppinger, 2012).

There is considerable evidence that decline in dopaminergic pathways may result in an impairment in decision-making abilities (Hämmerer and Eppinger, 2012).

Parkinson’s disease (PD) is a clinical condition of particular interest in this research field, because both the neuron loss and the pharmacological treatment affect dopaminergic transmission and influence the function of the fronto-striatal loop. A growing bulk of recent literature has documented the presence of feedback processing deficits in PD patients (Frank et al., 2004, 2007; Bódi et al., 2009; Kobayakawa et al., 2010; Kapogiannis et al., 2011), concurrently with the development of cognitive and behavioral deficits linked to the impulse control disorder spectrum (Poletti and Bonuccelli, 2012). The application of one of the most common decision-making tasks, i.e., the Iowa Gambing Task (IGT; Bechara et al., 1994), which does not offer the knowledge about the probabilities of certain outcomes and properly simulates the uncertainty of decision-making in the real life setting, in PD without dementia gave divergent results (Poletti et al., 2011; Dirnberger and Jahanshahi, 2013). Some studies reported no impairment (Thiel et al., 2003; Euteneuer et al., 2009; Poletti et al., 2010), but most of them showed worse performance in PD patients than healthy controls (Czernecki et al., 2002; Perretta et al., 2005; Mimura et al., 2006; Pagonabarraga et al., 2007; Kobayakawa et al., 2008, 2010; Gescheidt et al., 2012).

The role of dopaminergic drugs is also not completely clear, in that some studies documented no effect of the treatment on the IGT performance (Czernecki et al., 2002; Perretta et al., 2005; Kobayakawa et al., 2010), while other ones showed that patients were more impaired when treated (Cools et al., 2003; Euteneuer et al., 2009) and another report using a different gambling task found worse score in patients without medication (Brand et al., 2004). These findings appear to be in contrast with the view that the use of dopaminergic medication, in particular DA, instead of the neuronal loss, are responsible for impulse control disorders in PD (Weintraub and Nirenberg, 2013).

Finally, no significant difference was found in IGT results when comparing PD patients with and without dementia (Delazer et al., 2009); this finding is in keeping with the notion that executive dysfunction occurs early in the natural history of PD (Dirnberger and Jahanshahi, 2013).

The present study is aimed to shed some light in this field, and to overcome some limits and discrepancies of previous studies. To this aim, we explored one of the crucial aspects of decision-making ability, i.e., the outcome evaluation with IGT in medicated PD patients.

In addition to behavioral response, this is the first study to explore the brain correlates of feedback processing with electroencephalogram (EEG) and event related potentials (ERPs) recording in PD patients.

Monitoring the outcome of a decision evokes a large cortical response, which is mainly localized over central electrodes, and that can be separated in a feedback-related negativity (FRN) and a P300, with the former representing an early appraisal of feedback on a binary classification of good vs. bad outcome, and the latter resulting in a later top–down controlled evaluation process that is related to both the valence and the magnitude of the feedback (Gehring and Willoughby, 2002; Yeung and Sanfey, 2004; Toyomaki and Murohashi, 2005; Hajcak et al., 2006; Holroyd et al., 2006; Wu and Zhou, 2009; Cui et al., 2013; Ferdinand and Kray, 2013).

## MATERIALS AND METHODS

### PARTICIPANTS

Thirty participants were recruited: 15 (11 male) healthy subjects (age range 43–77 years; mean: 60.7, SD: 9.8) and 15 (10 male) PD patients (age range 41–73 years; mean: 61.4 years, SD: 9.6) participated in the study. The patients fulfilled diagnostic criteria for PD according to the PD Society Brain Bank Criteria (Hughes et al., 1992). PD patients had mean disease duration of 4.8 years (range of onset 1–14 years, SD: 3.4) and a mean estimated motor sub score of 8.9 (range 3–16, SD: 4) on the UPDRS part III (Fahn et al., 1987; Goetz et al., 2003). Patients were asked to continue taking their medication at the required time on the day of testing. Six patients received dopamine precursors (levodopa), three patients were receiving dopamine agonists, four received a monoamine oxidase inhibitor (MAOI), and two patients were taking a combination of levodopa and dopamine agonists. The average levodopa equivalent was 457 ± 122.7 mg. Healthy subjects and PD patients were matched for age, gender, education, and MMSE score (see **Table 1**) and for this reason the healthy subjects will be considered as control group. All participants gave signed informed consent after the purpose of the study and the protocol had been explained to them. The study was approved by the local ethics committee of the Department of General Psychology of the University of Padua.

**Table 1:** Means and standard deviations of matched demographical characteristics and MMSE score in PD patients and control group.

	PD patients *N* = 15	Control group *N*= 15	Test (df)	*p*-value
Age (years)	61.4 ± 9.6	60.7 ± 9.8	t_(28)_ = 0.245	ns
Gender	10 M	11 M	x^2^_(1)_ = 0.159	ns
Education (years)	8.7 ± 3.6	11.4 ± 4.3	t_(28)_ = –1.82	ns
MMSE score	28.3 ± 1.2	27.86 ± 1.5	t_(28)_ = 0.784	ns

*MMSE: Mini Mental State Examination (Folstein et al., 1975).*

### EXCLUSION/INCLUSION CRITERIA

Inclusion criteria for this study were participants with normal or corrected to normal vision. Exclusion criteria applied in the recruitment of the control group were the presence of neurological disease (any medical conditions associated with a head injury, epilepsy, stroke), reported history of psychiatric disorder or neurological disease and use of psychiatric and neurological medications.

Finally, for both patients and control group exclusion criteria were a Mini Mental State Examination score (MMSE; Folstein et al., 1975) < 24 and a Beck Depression Inventory score (BDI; Beck et al., 1961) < 14.

### MEASURES

#### Iowa gambling task

Decision-making was assessed using the Iowa gambling task (IGT; Bechara et al., 1994). This test was developed in the Iowa University to assess decision-making capacity in laboratory environment. Even if it was originally designed in analogical mode, in our study the IGT was implemented in a computerized version. The experiment ran with the E-Prime 2 software (Psychology Software Tools, Pittsburgh, PA, USA) installed on a personal computer equipped with a 17” monitor.

The task consisted in the presentation, on a computer screen, of four decks named A, B, C, and D. Each card in these decks can bring a win or a loss: participants were requested to gain as more as possible, choosing consecutively one card from any of the four decks, until the task shuts off automatically after 100 cards. The back of each deck looks the same, but they differ in composition. Decks A and B are considered disadvantageous, because they brought to big wins but also expensive losses, producing a net loss of 250€ in 10 cards. Deck C and D are considered advantageous decks because brought small wins, but smaller losses, causing a net gain of 250€ in block of 10 cards. The instructions given to the participants were the following: “ *in this screen you can see four decks, two are advantageous and two are disadvantageous. Each card of these decks can bring a win or a loss: the goal of this task is to win as much money as possible, and avoid losing money as much as possible, starting from a virtual budget of 2000€*.” Participants did not know the number of choices and, moreover, which were the advantageous or the disadvantageous decks. Participants saw on the screen the amount of money that they won or loose; this amount was updated after each choice.

#### EEG recording

While participants performed the IGT, the EEG was acquired from an array of 32 Ag/AgCl electrodes, through a Micromed electrode system. Electrodes were identified by brain hemisphere (odd numbers = left, even numbers = right) and general cortical zone (F = frontal, C = central, T = temporal, P = parietal, and O = occipital). 30 electrodes were mounted on an elastic cap, according to the International 10–20 system (Oostenveld and Praamstra, 2001). Left and right mastoids served as reference, while the vertical and horizontal eye movements were recorded with two electro-oculogram (EOG) electrodes, placed below and at the outer canthus of the left eye. The ground electrode was located at POz channel. Rating sample was 512 Hz, electrodes impedances were <5 kΩ and a digital band-pass filter from 0.1 Hz to 30 Hz was applied off-line.

#### Behavioral variables

The IGT performance was evaluated using more then one parameters. The first analysis has been conducted exploring the modal value concerning decks choices. The preferential choice for each subject of the two groups was calculated, and the values were submitted to a Chi square frequency analysis, to evaluate if the distribution of choice frequencies was the same in the two groups. To obtain the *learning* IGT scores, according to previous reports (Bechara et al., 1994; Fukui et al., 2005; Pagonabarraga et al., 2007; Kobayakawa et al., 2010) we subdivided the 100 selections into five blocks of 20 cards. For each block, the difference between the number of cards selected in advantageous decks (A and B) minus those chosen in disadvantageous ones (C and D) was calculated. In this way, five IGT scores were obtained for each participant, and the comparison between these values was considered as index of learning trend. In fact, increasing values of IGT score from the first to the last block indicate a preference for advantageous decks and the learning of the correct response strategy.

A *total* IGT score was finally calculated by means of the difference between overall advantageous choices minus overall disadvantageous choices. Pearson’s coefficient was calculated to correlate the *total* IGT score with clinical parameters as the disease’s duration, the motor UPDRS score and the age of onset. Group differences were investigated submitting *learning* IGT scores to a mixed model repeated ANOVA, with the factors group (patients and controls) and time (from the first to the fifth block). Bonferroni correction for multiple comparisons was applied.

#### ERPs data

EEG data were processed using EEGLAB (Delorme and Makeig, 2004). Epochs were locked to feedback onset and were 2000 ms long, between 1000 ms before and 1000 ms after feedback onset; the averaging procedure was performed separately for positive and negative feedbacks.

Non-significant differences were found comparing the number of epochs corresponding to positive and negative feedbacks in the two groups.

Artifacts correction was performed using independent components analysis technique (ICA; Makeig et al., 1996). Mean amplitude of three time windows was calculated at the midline electrodes Fz, Cz, and Pz, to measure P200 (150–250 ms), FRN (250–350 ms), and P300 (350–450 ms). These values were submitted to a mixed model repeated ANOVA, with the factors Interval (150–250 ms, 250–350 ms, and 350–450 ms) Site (Fz, Cz, Pz), Feedback type (win vs loss), and Group (PD patients vs Control group). Bonferroni correction for multiple comparisons was applied.

## RESULTS

### BEHAVIORAL RESULTS

Exploring the modal values of deck choices, calculated for each subject of the two groups, results showed that 66% of our patients preferred disadvantageous decks; only five patients (34%) preferred advantageous decks. On the contrary, the control group showed the opposite pattern: on 15 participants, the 80% preferred advantageous decks, while only 3 subject (20%) choose as preferential deck a disadvantageous one. The pattern of these choices was significantly different between patients and controls (χ^2^_(1)_= 0 6.65; *p* < 0.05).

The correlational analysis results on the *total* IGT score showed no significant correlations between the performance on the IGT and the disease’s duration, the age of onset and the motor UPDRS score (*p* > 0.05).

**FIGURE 1:**
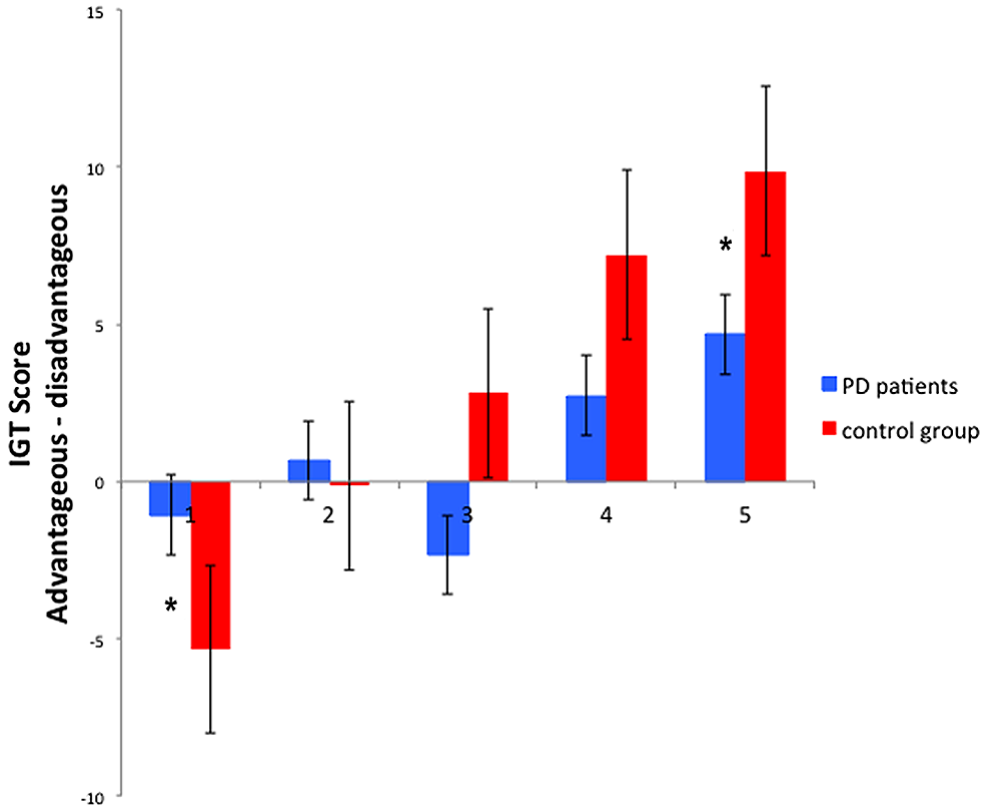
**Behavioral results of the Iowa gambling task: *learning* IGT scores in the five blocks, of PD patients and control group.** *Significant difference refers to a *p* value <0.05. Error bars represent standard errors.

Evaluating learning trend along time during the task, the ANOVA on *learning* IGT scores showed a main effect of Time [*F*(4,112) = 14.27, *p* < 0.001, $\eta_{p}^{2}$ = 0.338], showing that overall participants chose the advantageous decks more frequently in the last block compared to the first (*p* < 0.05). A significant Time^∗^Group interaction [*F*(4,112) = 3.75,* p* < 0.01, $\eta_{p}^{2}$ = 0.118], show that despite a better performance of PD patients in the first block (*p* < 0.05), PD patients had a significantly lower *learning* IGT score, respect to the control group, in the fifth block (*p* < 0.05; see **Figure 1**).

### ERPs RESULTS

**FIGURE 2:**
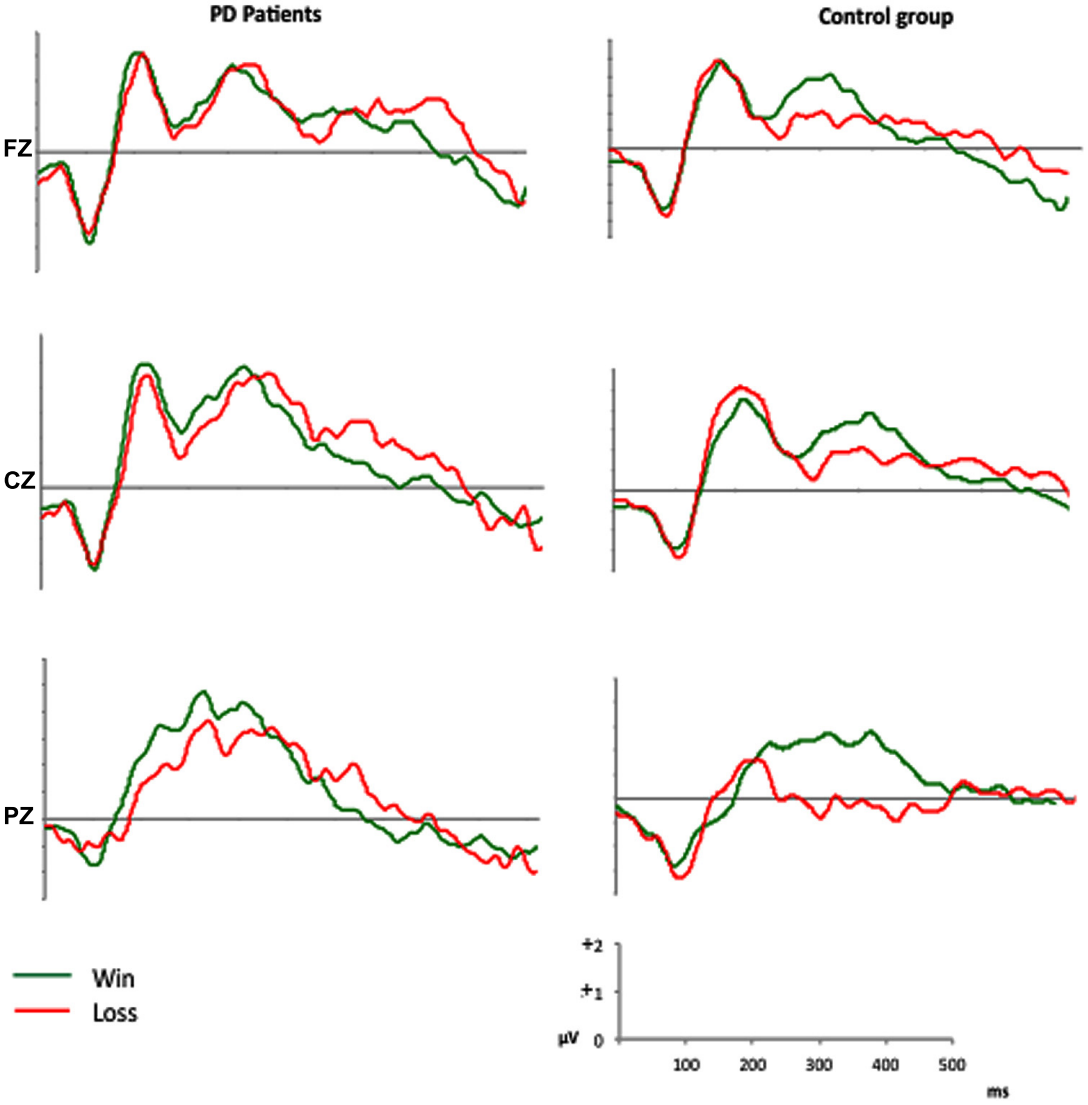
**Grand average ERPs in the anterior (Fz), central (Cz), and parietal (Pz) sites**.

The feedback-locked ERPs of both groups are displayed in **Figure 2**.

**FIGURE 3:**
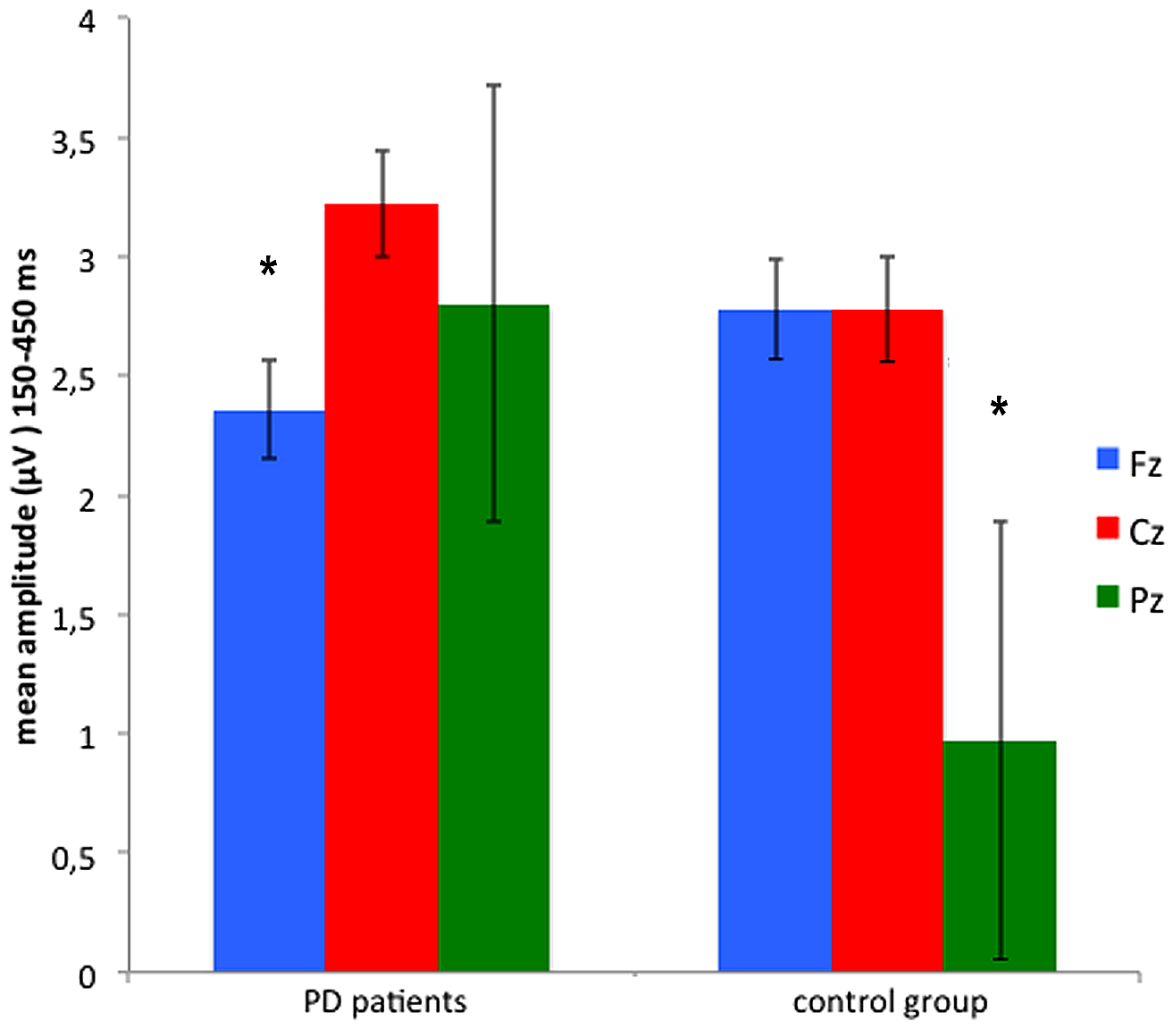
**Mean amplitude recorded in the time window between 150 and 450 ms from anterior (Fz) central (Cz) and parietal (Pz) sites.** *Significant difference refers to a *p* value <0.05. Error bars represent standard errors.

The analysis of the mean amplitude recorded in the three time intervals after feedback onset (150–250 ms, 250–350 ms, and 350–450 ms) and at the midline electrodes Fz, Cz, and Pz, showed main effects of Site [*F*(2,56) = 4.46, *p* < 0.05, $\eta_{p}^{2}$ = 0.137] and Feedback type [*F*(1,28) = 7.07, *p* < 0.05, $\eta_{p}^{2}$ = 0.202]: mean activity between 150 and 450 ms after feedback onset has higher amplitude at Cz (3.00 μV), comparing with Fz (2.57 μV), and Pz (1.89 μV). In addition, the ERPs amplitude was greater after positive feedbacks (2.96 μV) then negative ones (2.02 μV). The difference between positive and negative feedbacks was significant between 250 and 450 ms, as indicated by the Feedback^∗^Time interaction [*F*(2,56) = 3.16, *p* < 0.05, $\eta_{p}^{2}$ = 0.102]. Site^∗^Group interaction [*F*(2,56) = 4.53, *p* < 0.05, $\eta_{p}^{2}$ = 0.139] and subsequent *post hoc* comparisons, indicated that PD patients had a lower (*p* < 0.005) amplitude at frontal site (Fz) compared with central site (Cz), and a comparable amplitude at central (Cz) and parietal (Pz) sites. On the contrary, control group showed a significantly lower activity (*p* < 0.05) at the parietal site (Pz), comparing with central (Cz) and frontal (Fz) ones (see **Figure 3**).

The Site^∗^Feedback type interaction was also significant [*F*(2,56) = 4.0, *p* < 0.05, $\eta_{p}^{2}$ = 0.126], indicating significant differences between positive and negative feedback-evoked responses in Fz and Pz.

**FIGURE 4:**
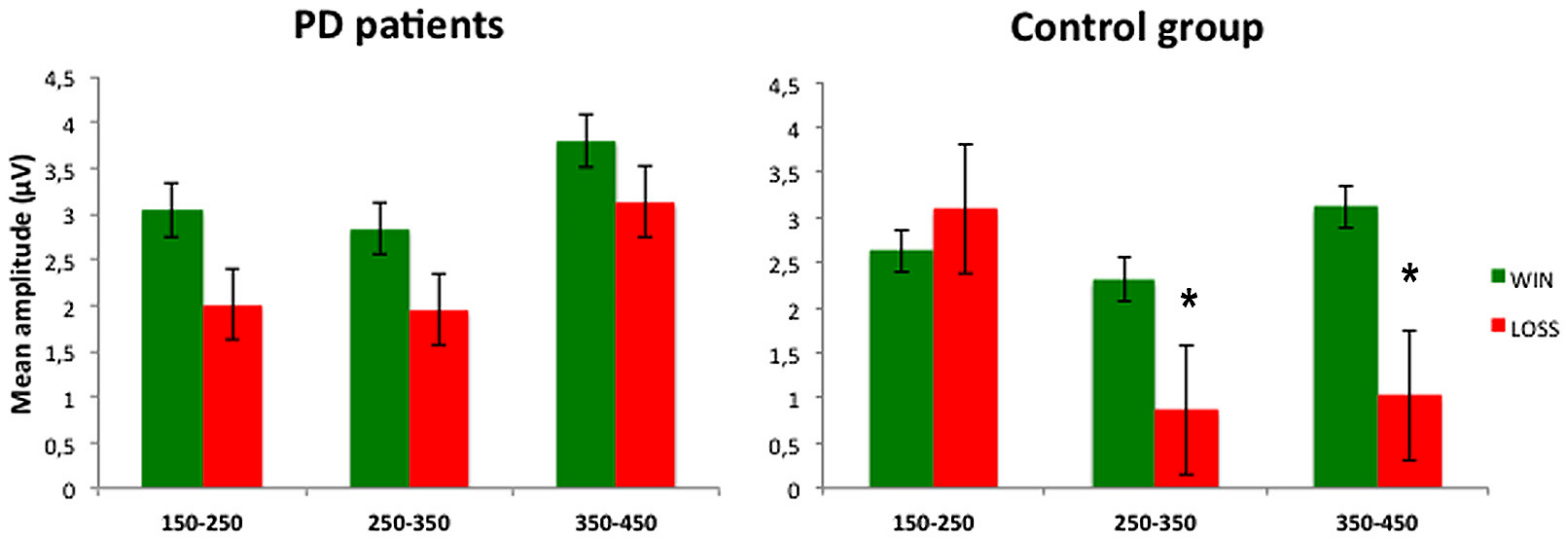
**Mean amplitude recorded in three time windows (150–250 ms; 250–350 ms; 350–450 ms) for win and loss conditions.** *Significant difference refers to a *p* value <0.05. Error bars represent standard errors.

Finally, a significant interaction Feedback^∗^Time^∗^Group [*F*(2,56) = 5.21, *p* < 0.01, $\eta_{p}^{2}$ = 0.157] indicated that PD patients and control group presented different feedback-evoked responses. *Post hoc* comparisons specified that in the control group the mean amplitude, of both the time windows 250–350 and 350–450 ms, was significantly different after positive and negative feedbacks (*p* < 0.05). On the contrary, in PD patients, non-significant differences between feedback-evoked responses were found (see **Figure 4**). Furthermore, *post hoc* comparisons also revealed that PD patients and control group showed different ERPs responses recorded in PZ channel after negative feedback, specifically in the time window between 250 and 350 ms (*p* < 0.05).

## DISCUSSION

In the current study we examined behavioral responses and their neural correlates during the IGT (Bechara et al., 1994), a task that simulates an uncertain decision-making situation, in a sample of non-demented and non-depressed PD patients on therapy. Our aims were to add evidence in this topic, given the discordant findings from previous reports, and to focus on the cortical responses during feedback processing using ERPs (Fang et al., 2009). To the best of our knowledge, this is the first study to explore ERPs during IGT in PD.

The present results indicate that medicated PD patients had a lower performance on the IGT compared to a control group of healthy subjects. While controls showed learning process during the task (i.e., they progressively chose more frequently the advantageous decks across the experimental blocks), PD patients preferred disadvantageous decks and, more interestingly they did not ameliorate across the task. ERPs findings suggest that the problem with learning a strategy during the task is secondary to abnormal feedback processing in PD patients. ERPs behave differently according to the feedback valence in normal controls, in that they did not differ in voltage amplitudes in the early window (150–250 ms), but they were significantly larger to wins vs. losses in the windows that correspond to the FRN (250–350 ms) and P300 components (350–450 ms), respectively. At variance, no difference was found for any time window in patients according to the valence of the feedback. Furthermore, scalp topography of ERPs was shifted posteriorly in PD patients when compared to controls.

In accordance with previous studies (Czernecki et al., 2002; Perretta et al., 2005; Mimura et al., 2006; Pagonabarraga et al., 2007; Kobayakawa et al., 2008, 2010; Gescheidt et al., 2012), our behavioral data indicate a difficulty to learn and follow a successful strategy to improve their performance and a preference for disadvantageous decks in PD patients.

In keeping with previous reports (Pagonabarraga et al., 2007), IGT performance was not correlated with age of onset, PD duration or motor severity, indicating that impairment in decision-making, and motor performance are unrelated to each other. This finding is in agreement with the clinical evidence of executive dysfunction despite very good motor performance in some PD patients. Any possible effect of dementia was ruled out by the inclusion of non-demented patients in our study.

The analysis of feedback-related ERPs offered some insight on the brain mechanisms underscoring the abnormal IGT performance in our PD patients. To better explore the different stages of feedback processing, we analyzed ERPs across three windows.

The first window (150–250 ms) comprised the very early component, which is named P200 and is more marked in the frontal regions (Polezzi et al., 2008; Schuermann et al., 2012). The second window (250–350 ms) was focussed on the FRN, which reflects the early feedback appraisal on a binary good vs. bad classification according to the subject’s expectation (Schuermann et al., 2011) and whose source is located in the medial frontal cortex (Gehring and Willoughby, 2002). The third window (350450 ms) explored the P300 that is related to a more complex feedback evaluation reflecting the allocation of motivational and attentional resources and shows the larger amplitude in the central and parietal regions (Schuermann et al., 2011; Cui et al., 2013).

We found that, while ERPs amplitudes were significantly larger for positive vs. negative feedback in normal controls, this difference was absent in PD patients both for the FRN and the P300 time windows. At variance the behavior of the P200 window was the same in the two groups. These findings suggest that PD patients are not able to separate feedbacks according to their valence and that these abnormalities occur across different stages of feedback evaluation.

The FRN is an index of the violation of the expectations of the subject rather than of the absolute valence of the feedback and is generated in the ACC (Holroyd et al., 2006; Oliveira et al., 2007; Jessup et al., 2010; Alexander and Brown, 2011).

At variance, the P300 is a more complex phenomenon that reflects the valence of the feedback, contributes to performance monitoring and behavioral adaptation (Ferdinand and Kray, 2013) and is influenced by attention and working memory updating (Donchin and Coles, 1988; Polich, 2004, 2007). The P300 typically shows the *positivity effect* (i.e., a larger amplitude to positive than negative feedback), which is supposed to reflect a positive feedback as more task relevant, because it signals that the intended goal has been achieved (Bellebaum and Daum, 2008; Ferdinand and Kray, 2013).

It is not surprising that the P200 component did not change in relation to positive vs. negative feedback in patients and controls, as this ERP component has been found to be related to the unpredictability of outcomes, rather than their valence (Polezzi et al., 2008).

Behavioral and ERPs abnormalities in PD patients might be explained in light of current knowledge of the functional anatomy underlying IGT performance. A brain network including the amygdala, the orbital PFC (oPFC), the ACC, the dorsolateral PFC (dlPFC) as well as ventral and dorsal striatum is critically involved in decision-making (Delazer et al., 2009). FRN changes might be ascribed to abnormal activity in the ACC (Gehring and Willoughby, 2002), which plays a major role in this network.

Two hypotheses may be set forth to explain in more details the mechanisms underlying our findings. Sensitivity to negative stimuli has been associated with the integrity of the amygdala (Bechara et al., 1999), which might be involved in the presymptomatic stage of PD according to Braak’s neuropathological staging (Braak et al., 2006). This first hypothesis is in keeping with previous reports of abnormal electrodermal responses during IGT in PD similar to that of amygdala-damaged patients (Kobayakawa et al., 2008) especially to negative feedback (Euteneuer et al., 2009).

The second hypothesis stems from a neurobiologically based computational model, which indicates that negative feedback triggers dopamine dips in the basal ganglia indirect pathway leading to No-Go-learning in decision-making (Frank et al., 2004). Dopaminergic drugs might impair learning from negative feedback, because they block the physiological effect of dopamine dips (Frank et al., 2004; Euteneur et al., 2007). This model would fit well with the P300 abnormalities in PD patients along with the difficulties in learning a strategy during IGT.

The relative dopamine sparing of the circuit linking the ventral striatum to the oPFC in comparison to that connecting the dorsal striatum and the dlPFC would lead to normalization of the function of the latter with a relative dopaminergic overdose in the former resulting in an impairment of decision-making tasks such as the IGT (Perretta et al., 2005).

This view is supported by functional neuroimaging studies documenting a dysfunction of the non-motor loop linking the oPFC, and the ACC to the ventral striatum (Thiel et al., 2003), more evident after negative feedback, despite a preservation of the dlPFC and the amygdala (Gescheidt et al., 2013) and by similar findings of abnormal IGT learning in patients with lesions restricted to the oPFC (Bechara et al., 2000).

Our findings seem to support the second hypothesis: the significant difference found in the ERPs response evoked by negative feedback supported the assumption that dopaminergic medication specifically affects the processing of negative stimuli. The role of dopaminergic drugs in impairing the response to negative feedback is further supported by previous studies on IGT (Perretta et al., 2005; Pagonabarraga et al., 2007; Kobayakawa et al., 2008; Delazer et al., 2009) and reward-learning (Bódi et al., 2009) on medicated PD, as well as by the normal IGT performance in *de novo* non-medicated PD patients (Poletti et al., 2010).

In accordance with previous reports, we found no difference between levodopa and dopamine agonists (Pagonabarraga et al., 2007; Kapogiannis et al., 2011), but the small number of subjects and the use of multiple medications in some patients may have contributed to this negative finding.

The absence of any effect of drug class appears to be in contrast with the notion that pathological gambling is more frequent in patients with dopamine agonists (Weintraub and Nirenberg, 2013).

Despite the similarity between the present IGT abnormalities in PD and those found in pathological gamblers (Goudriaan et al., 2005), none of our patients presented symptoms of gambling.

However, PD patients frequently show impulsive behavior and the present ERP abnormalities are similar to those found in a wider spectrum of neuropsychiatric conditions that share the presence of impulse control disorder and include borderline personality disorder (Schuermann et al., 2011), attention-deficit/hyperactivity disorder and bipolar disorder (Ibanez et al., 2012), and problem gambling (Oberg et al., 2011).

The exclusion of depressed patients ruled out a possible contribution of the dysfunction of serotoninergic pathways, which contribute to learning (Gleichgerrcht et al., 2010), in our patients.

While ERPs were larger on more anterior sites (Fz, Cz) in comparison to Pz in normal controls, they appeared to be significantly smaller in Fz in patients indicating a posterior shift in PD. The anterior-to-posterior gradient of ERPs is a well-known finding in older adults and has been interpreted in terms of compensatory resource allocation (Reuter-Lorenz and Lustig, 2005; Adrover-Roig and Barceló, 2010; Daffner et al., 2006, 2011; Ferdinand and Kray, 2013). At variance, the posterior shift of ERPs in PD patients suggested a different recruitment of neural resources and could be interpreted as a failure of PD patients in this compensatory mechanism, probably due to PFC dysfunction. A similar shift toward posterior electrodes was found with early somatosensory evoked potentials (SEPs) that documented a marked reduction of frontal N30 component and indicate a frontal dysfunction in PD patients (Garcia et al., 1995; Bostantjopoulou et al., 2000).

In conclusion, our behavioral results confirmed worse IGT performance in medicated PD patients and ERPs data offered some insight on the underlying mechanisms pointing to PFC dysfunction related to dopaminergic treatment. These abnormalities are in line with the growing literature about changes in feedback processing in this condition (Frank et al., 2004, 2007; Bódi et al., 2009; Kobayakawa et al., 2010 Kapogiannis et al., 2011) and may contribute to cognitive and behavioral problems related to impulse control disorder and to the impairment in every-day decisions that is common in PD patients.

## Conflict of Interest Statement

The authors declare that the research was conducted in the absence of any commercial or financial relationships that could be construed as a potential conflict of interest.
